# Deep Sequencing Analysis of Hepatitis C Virus Subtypes and Resistance-Associated Substitutions in Genotype 4 Patients Resistant to Direct-Acting Antiviral (DAA) Treatment in Egypt

**DOI:** 10.3390/ijms262110649

**Published:** 2025-10-31

**Authors:** Damir Garcia-Cehic, Asmaa Mosbeh, Heba A. Gad, Asmaa Ibrahim Gomaa, Marta Ibañez Lligoña, Josep Gregori, Sergi Colomer-Castell, Carolina Campos, Francisco Rodriguez-Frias, Juan Ignacio Esteban, Mohamed S. Kohla, Mohamed Helmy Abdel-Rahman, Josep Quer

**Affiliations:** 1 Department of Radiation Oncology, College of Medicine, University of Cincinnati, Cincinnati, OH 45267, USA; garciadh@ucmail.uc.edu; 2 Liver Diseases-Viral Hepatitis, Liver Unit, Vall d’Hebron Institut de Recerca (VHIR), Vall d’Hebron Barcelona Hospital Campus, 08035 Barcelona, Spain; marta.ibanez@vhir.org (M.I.L.); josep.gregori@gmail.com (J.G.); sergi.colomer@vhir.org (S.C.-C.); carolina.campos@vhir.org (C.C.); frarodri@gmail.com (F.R.-F.); nyanyoesteban@gmail.com (J.I.E.); 3 Centro de Investigación Biomédica en Red de Enfermedades Hepáticas y Digestivas (CIBERehd), Instituto de Salud Carlos III, 28029 Madrid, Spain; 4 Department of Pathology, National Liver Institute, Menoufia University, Shibin el Kom 32511, Egypt; asmaa.abdelmaksoud@osumc.edu; 5 Sustainable Sciences Institute Collaborative Research Center, Menoufia University, Shibin el Kom 32511, Egypt; hebagad306@gmail.com (H.A.G.); dr_mohamedsamy@yahoo.com (M.S.K.); 6 Molecular Diagnostic Unit, New Kasr El-Ainy Teaching Hospital, Cairo University, Giza 12613, Egypt; 7 Department of Hepatology, National Liver Institute, Menoufia University, Shibin el Kom 32511, Egypt; aibrahim@liver-eg.org; 8 Medicine Department, Universitat Autònoma de Barcelona, 08193 Bellaterra, Spain; 9 Department of Ophthalmology, The Ohio State University, Columbus, OH 43210, USA; 10 Division of Human Genetics, The Ohio State University, Columbus, OH 43210, USA; 11 Biochemistry and Molecular Biology Department, Universitat Autònoma de Barcelona, 08193 Bellaterra, Spain

**Keywords:** HCV subtypes, NS5A, RAS, DAA, deep sequencing

## Abstract

Egypt has the highest global prevalence of hepatitis C virus (HCV), with genotype 4 (G4) in over 94% of cases. Direct-acting antivirals (DAAs) yield sustained virologic response (SVR) rates above 95%. Second-generation DAAs are recommended for patients with virological failure, achieving over 90% eradication. This study aimed to classify and evaluate the pattern of HCV resistance-associated substitutions (RASs) in patients who failed DAA treatment in Egypt. A total of 1778 chronically infected HCV patients from Egypt’s Nile Delta were enrolled (2016–2018). Among them, 37 relapsed, and high-quality serum samples from 22 patients were available, including 6 cases with pre- and post-treatment samples. Next-generation sequencing (NGS) was performed for HCV subtyping and RAS identification. Among the 22 analyzed cases, 21 (95.4%) were G4: 11 were classified as subtype G4a, seven G4o, and three G4m. One patient (4.5%) was identified as G1g. One case shifted from G4a pre- to G4o post-treatment, suggesting reinfection. The RAS pattern in rare G4 subtypes (G4m/G4o) differs from the G4a subtype. The combination of L28M/L30S mutations was detected in 8/11 G4a samples; in contrast, RASs in G4o were characterized by T30S or Y93C/H/N/S substitutions. Notably, some substitutions identified as RASs may represent fixed polymorphisms in regional viral populations, such as those in Egypt’s Nile Delta. HCV subtypes significantly influence the RAS pattern, particularly within the NS5A region, after DAA-treatment failure. The RAS pattern differs among G4 subtypes, particularly in rare ones, predisposing patients to resistance and underscoring the importance of NGS in regional populations to optimize treatment strategies.

## 1. Introduction

Hepatitis C virus (HCV) infection is a public health concern in many countries, and still a major cause of liver-related morbidity and mortality despite the advances in the treatments with combination of direct-acting antivirals (DAAs). Globally, approximately 1.5 million patients are newly infected every year, with a global prevalence of 0.8% in the general population. Currently, at least 58 million patients are chronically infected with HCV, leading to significant mortality due to cirrhosis and hepatocellular carcinoma [1].

Although DAAs have revolutionized HCV therapy with high rates of sustained virologic response [2], therapeutic failure still occurs in a small proportion of patients, almost always due to the selection of resistance-associated substitutions (RASs) [3]. The presence and type of RASs determine the effectiveness of subsequent rescue regimens, as virologic failure leads to an altered viral landscape dominated by resistant variants, complicating future treatment options [4,5].

In 2015, the rate of HCV infection among adults in Egypt was 7%, the highest in the world, and accounted for 7.6% of the country’s mortality [6]. To address this significant public health challenge, Egypt established a national HCV control strategy and developed an HCV prevention and treatment system [7]. In partnership with GILEAD, Egypt introduced a countrywide campaign to eliminate hepatitis C, resulting in testing of over 60 million people and treatment of more than 4.1 million people, aiming at providing treatment to over 250,000 chronically infected patients annually [8,9]. Most of these treatments were based on Sofosbuvir-containing regimens (targeting the NS5B polymerase) combined with NS5A inhibitors such as Daclatasvir, Ledipasvir, or Ombitasvir. Retreatment strategies for HCV are particularly hampered in cases of multidrug resistance, which challenges the possibility to switch DAA class [3,10], leading healthcare providers to prescribe rescue treatments based on these RASs [5,10,11,12,13,14,15,16]. After treatment failure, RASs in non-structural proteins NS3 and NS5B often become undetectable within months after stopping therapy, while NS5A RASs can persist for years [10] and negatively impact retreatment outcomes in patients previously treated with NS5A inhibitors [10,11]. Consequently, resistance-associated substitutions (RASs) in the NS5A region are the most likely to emerge in cases of virologic failure.

A systematic review of HCV genotypes in Egypt revealed limited diversity considering genotypes, with genotype 4 (G4) being dominant, with 92.5% of cases (12.1% subtype G4a and 82.3% unknown G4 subtypes). G1 accounted for 3.6% of cases and mixed infections were present in 3.2% of cases [6]. Globally, HCV-G4 accounts for 8–16.8% of all HCV cases, making it the third most common genotype, especially in lower-income countries [12,13].

Despite this apparent predominance, distinguishing among G4 subtypes (such as G4a, G4d, and G4o) is essential, since each exhibit characteristic mutations that may influence the natural prevalence of polymorphisms, the genetic barrier to resistance, and ultimately the response to DAAs. Nevertheless, there is a lack of information about the efficacy of the DAAs on different G4 subtypes other than G4a.

This study aimed to characterize and identify resistance-associated substitutions (RASs) and potential mutational patterns related to specific subtypes of HCV genotype 4. This approach seeks to improve the understanding of subtype-related resistance mechanisms and their impact on treatment outcomes.

## 2. Results

A total of 9,296,676 reads passed quality controls, with the number of reads per amplicon and sample ranging from 3824 to 244,682 (Supplementary Table S1). Twenty-two patients (cases) were included in the study, with six providing both pre- and a post-treatment samples, increasing the total number of NGS-subtyped samples to 28.

Among the 22 patients who experienced treatment failure with DAA-based regimens, 21 (95.4%) were identified with HCV genotype 4, and one (4.5%) with HCV genotype 1. High-resolution HCV subtyping using NGS revealed that 11 out of the 22 (50%) were classified as subtype G4a, seven (31.8%) as G4o, and three (13.6%) as G4m. The single genotype 1 was classified as G1g.

**Figure 1:**
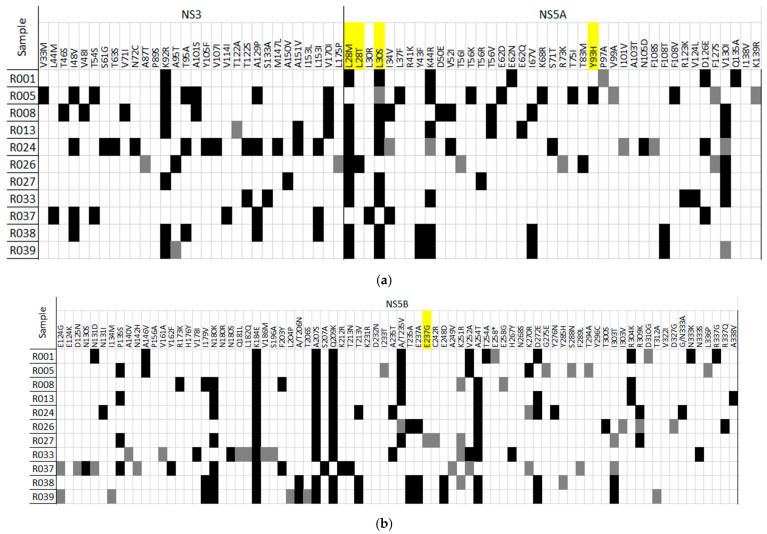
(**a**) G4a post-treatment samples substitutions detected at targeted regions from the NS3 and NS5A. In yellow, substitutions corresponding to G4 RASs according to Sorbo MC et al. [14]. In black, substitutions represented at the consensus sequence and in grey, mutations detected as minor variants. (**b**) G4a post-treatment samples substitutions detected at targeted region from the NS5B. In yellow, substitutions corresponding to G4 RASs according to Sorbo MC et al. [14]. In black, substitutions represented at the consensus sequence and in grey, mutations detected as minor variants.

**Figure 2:**
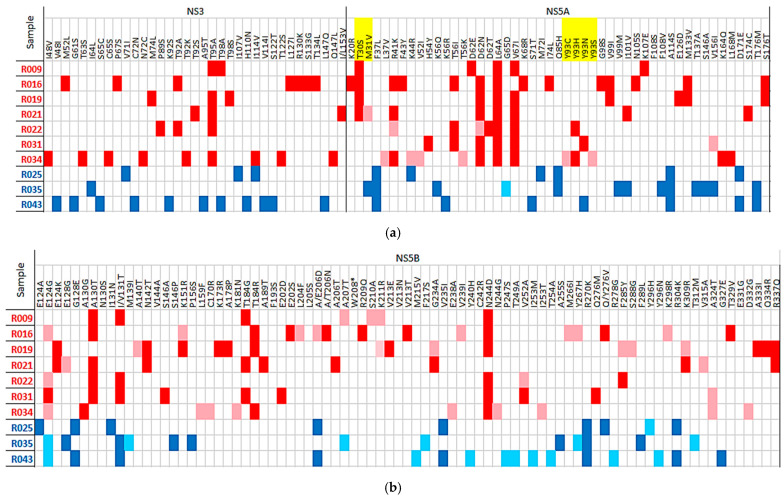
(**a**) G4m (blue) and G4o (red) post-treatment samples substitutions detected at targeted regions from the NS3 and NS5A. In yellow, substitutions corresponding to G4 RASs according to Sorbo MC et al. 2018 [14]. Minor variants are represented with a light color corresponding to subtype. (**b**) G4m (blue) and G4o (red) post-treatment samples substitutions detected at targeted region from the NS5B. In yellow, substitutions corresponding to G4 RASs according to Sorbo MC et al. [14]. Minor variants are represented with a light color corresponding to subtype.

**Figure 3:**
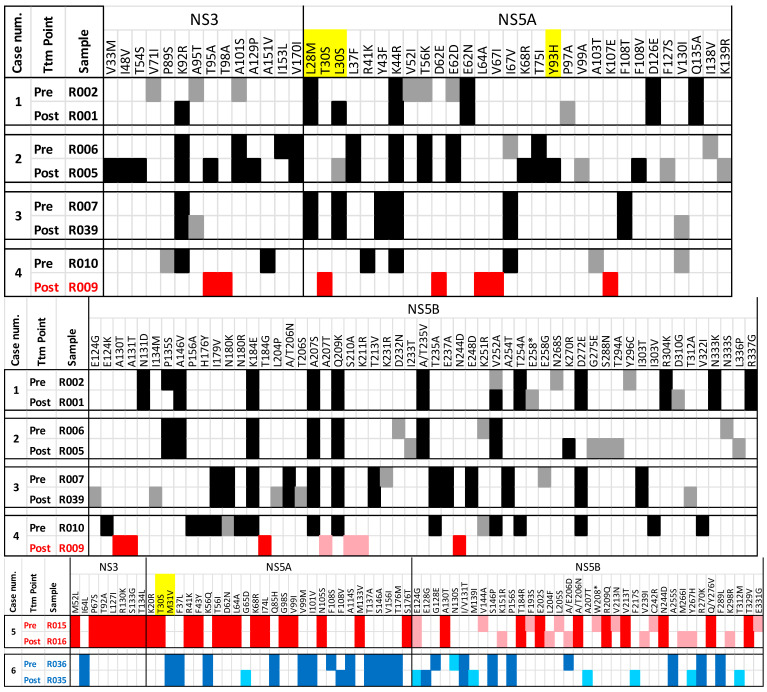
Pre- and post-treatment cases substitutions comparison. In yellow, substitutions corresponding to G4 RASs according to Sorbo MC et al. [14]. Minor variants are represented with a lighter color corresponding to subtype: black (G4a), red (G4o), and blue (G4m).

Raw data from all patients and targeted regions have been submitted to the GenBank Sequence Read Archive database with the project number “PRJNA1036116”. A full report including all substitutions is provided in Figure 1, Figure 2 and Figure 3, Table 1 and Table S1.

At treatment failure, all viral genomes corresponding to G4a samples exhibit at least one mutation at the amino acid positions 28 and 30 of the NS5A region. All the observed substitutions have been previously associated with resistance to DAAs (Figure 1a,b). Substitution L28M was present in 8 out of 11 G4a samples (72.7%), conferring resistance to Daclatasvir (DCV) (fold-change (FC) = 10), Elbasvir (EBR), and Ledipasvir (LDV) (FC = 100). Additionally, L30S was detected in 9 out of 11 (81.8%) and confers resistance to EBR (FC = 4). The presence of an S at position 30 of NS5A region, independently of the original amino acid, has also been associated to resistance to DCV (FC = 150–157) and LDV (FC = 100) [14]. Interestingly, the combination of RASs L28M + L30S were identified in 8 out of 11 cases (72.7%), and their co-occurrence in the same viral genome confers a higher resistance to LDV treatment (FC = 5000). Of note in sample R005, the RAS L30S was detected in a minority of sequences (4.6%), but we also detected RAS Y93H. Combination of RASs L30S and Y93H leads to more resistance to LDV (FC = 900,000). Alone, the Y93H substitution confers resistance to DCV (FC = 45–169), EBR (FC = 7.5), LDV (FC = 1000), and OMB. Sample R037 showed the substitution 30R, that has been associated with resistance in G4a samples to DCV (FC = 10), LDV (FC = 50), and VEL (FC = 2) [14].

Since there is limited information on RASs in genotypes 4 other than G4a, we relied on DAA-RAS data for G4a and G4d as references to analyze substitutions found in G4o and G4m subtypes (Figure 2a,b). Except for sample R025 from G4m, which did not exhibit any known RASs among the three regions analyzed, all other samples showed at least one RAS in the NS5A targeted region. Among the G4o samples, we identified two distinct groups: one with samples showing only T30S (4 out of 7), and the other with samples showing substitution C/H/N/S at amino acid Y93 (3 out of 7). The remaining two G4m samples had at least one RAS in the NS5A targeted region; one sample had the RAS M31V, while the other has the RAS Y93N.

In all G4 samples, no confirmed RASs were found in the other two-targeted regions (NS3 and NS5B). The only exception was sample R027 (G4a), where the RAS E237G at the NS5B region was detected as a minor variant, representing only 1%, 2491 reads of a total of 244,682 reads.

For the G1g sample, RAS Q30R (present at a frequency of 79.6%) was detected at the NS5A region, and no other RASs were found at any other region, based on comparison with validated data for G1a and G1b.

When comparing cases with both pre- and post-treatment samples, we can rule out case number 4 (samples R010 and R009) as being the same infection, as high-resolution HCV subtyping reveals different subtype classifications: G4a in the pre-treatment sample and G4o in the post-treatment sample, suggesting that this is not a case of relapse, but a new infection. This is further supported by the lack of correlation in the substitution’s profiles between the two samples. Therefore, sample R010 will not be considered the naïve sample corresponding to the relapse case represented by R009.

Except for case number 2, which was treated with PTV/r/OMB/RBV for 24 weeks, all other cases were treated with DCV/SOF with or without RBV for 12 weeks (Table 1). Regardless of the treatment received, RASs associated with antivirals used for NS5A region were prevalent in all cases: L28M, L/T30S, and M31V to Daclatasvir (DCV) and Y93H to Ombitasvir (OMB), even in those cases where RAS was not detected due to a very low frequency at the pre-treatment sample viral population, like in cases 1 and 2.

Interestingly, in 2 out of 22 treatment failures (R037 G4a and R025 G4o), no RASs were detected in any of the three targeted regions. Several explanations are discussed at the end of next section.

Moreover, in post-treatment samples from patients infected with G4a, several substitutions were detected in a majority of cases (ranging from 6 to all 12 patients), with a frequency of 50% or higher, and in many cases, reaching 100% (Table 2a). Interestingly, some of these substitutions, such as L28M and L30S, which have previously been identified as RASs, were found in 8 and 7 out of 12 patients, respectively, after treatment (Table 2b). Notably, L28M alone, as well as the combined L28M + L30S substitutions, were already present in pre-treatment samples (Supplementary Table S1) from patients R002 and R007, respectively, suggesting that these RASs might be prevalent in G4a patients living in the Nile Delta Region in Egypt.

This indicates that, in addition to the mentioned RAS, other substitutions may need to be considered as polymorphisms specific to this geographic region.

## 3. Discussion

Recent guidelines recommend a combination of second-generation DAAs for patients with virological failure after prior DAAs treatment, with virological cure rates above 98% [15]. However, in resource-limited settings, due to the high cost of these regimens, alternative approaches should be considered. This could involve first-generation DAA regimens, in addition to Sofosbuvir and Ribavirin, for extended durations [16]. The selection of this rescue therapy should be based on the RASs identified during the initial DDA therapy, making resistance testing a cornerstone of personalized treatment strategies to optimize responses.

The use of high-resolution methodologies, such as deep sequencing, facilitates accurate subtype classification [17], and identification of RASs in patients who experienced virological DAA-treatment failure, thereby guiding successful retreatment strategies [3].

As expected, the most prevalent HCV subtype among our Egyptian patients was G4. Notably, half of the patients were classified as subtype G4a, while the other half belonged to rare subtypes G4o and G4m, for which little information has been published.

In our cohort of 22 patients, no RASs were detected in the NS3 region, and only one minor variant was found in NS5B. Consistent with other studies [18], RASs predominantly occurred in the NS5A across G4 subtypes, with subtype-specific patterns emerging post-treatment, highlighting the need for continued RAS monitoring in rare subtypes. The finding of RAS mutations predominantly in NS5A is particularly relevant in our cohort of genotype 4 patients treated with DCV, as most of these mutations confer resistance to DCV. Furthermore, this observation may explain why, when treatment involves two inhibitors and one is compromised by the presence of RASs in circulating virions, the therapeutic regimen effectively depends on a single active inhibitor and treatment is predisposed to failure.

Many substitutions appeared in 50% or more (including 100%) of the reads for most patients. It is important to clarify that these substitutions are reported in comparison to GenBank reference sequences, which may have been sourced from different geographic locations. In this study, we specifically studied a cohort of patients from the Nile Delta Region in Egypt. For G4a samples, it is possible that some amino acid positions we identified as substitutions may, in fact, be polymorphisms that have become fixed in the viral population of this region, as previously shown for G1a, G1b, and G3a in other locations [19,20,21,22]. In consequence, if these mutations are associated with antiviral resistance, it suggests that patients carrying these specific variants could be more likely to show resistance to the antivirals commonly used to treat G4a infections that lack these particular resistance-associated substitutions. This explanation may also apply to the rare G4 variants (G4o and G4m) that have limited or no prior reports in the literature, as some mutations could confer cross-resistance between different subtypes. Therefore, it is crucial to emphasize that NGS should be performed in local and/or regional populations when designing global treatment strategies, especially because any mutant present at frequencies greater than 1% could be clinically relevant for treatment outcomes [22]. Alternatively, some treatment recommendations based on global data may not be universally effective, as the circulation of certain variants in specific regions might render the virus in those areas resistant to particular antivirals.

In our study with patients that had failed DAAs, very few RASs have been reported in the NS5B region; only one minor variant (E237G) associated with SOF resistance was found in one patient. Actually, HCV G4 clearly shows very few RASs at baseline in the NS5B region [23]. Since most NS5B mutations reduce viral fitness, RASs are often outcompeted by wild-type variants after treatment ends, reverting to undetectable levels with population sequencing [24,25]. However, in full-length HCV genotype 4a infectious culture systems, it has been shown that the principal resistant substitution to SOF, NS5B-S282T, showed relatively high fitness and stability [26], suggesting that more studies with human plasma samples collected at the end of treatment (EOT) would be of great interest to clarify this issue and to assess the reversion of such NS5B-RASs after several weeks after OFT. It is important to note that the post-treatment samples included in this study were collected 12w after end-of-treatment, which aligns with international guidelines recommending HCV-RNA testing at this time point to determine whether sustained virological response has been achieved or if relapse has occurred. During these 12w, low-fitness variants gradually decline in frequency as they are outcompeted by higher-fitness variants, potentially becoming too rare to be detected even with high-resolution sequencing methods. However, if the mutant (i.e., the sequence carrying the RAS) persists, any rescue treatment using the same antiviral to which the mutant is resistant is doomed to fail [27]. Hence, as previously suggested, it is crucial to perform deep sequencing at the end of treatment to detect these variants before they become too rare to be detected [27].

This study had several limitations, including the absence of pre-treatment samples for comparison with post-failure samples, small sample size (particularly for rare subtypes), selection bias (40% exclusion rate), and limited sequencing coverage restricted to specific genomic regions. Additionally, we were unable to collect follow-up samples to study whether RAS mutations observed at failure were fixed or transient. In two out of 22 treatment failures, no RAS was detected in any of the targeted regions. There are several alternative explanations. One possibility is that the resistance substitution exhibits very low fitness and, although selected during therapy, viral mutants carrying this substitution are not maintained as predominant in the quasispecies after treatment stopping, making them undetectable even by deep sequencing. Another explanation could be that treatment failure involves mutations at sensitive positions that have not been previously reported, such as residues affecting protein structure or positions causing allosteric effects that alter drug binding efficiency. Finally, we recently reported that the QS maturity is directly associated with antiviral resistance, even in the absence of resistance-associated substitutions. In this context, QS maturity evolves continuously during chronic HCV infection, towards a flat-like structure that has been linked to higher rates of antiviral treatment failure, even in the absence of RASs in the viral genomes [28].

## 4. Materials/Patients and Methods

A total of 1778 patients with chronic HCV infection were enrolled in a biobanking study at the National Liver Institute Sustainable Sciences Institute Collaborative Research Center (NLISSICRC), National Liver Institute, Menoufia University in Egypt, between 2016 and 2018 from the Nile Delta Region (Egypt). Among these patients, 37 experienced DAA-treatment failure, evidenced by detectable HCV RNA 12 weeks after treatment (SVR12), confirmed by quantitative PCR (qPCR) using the Artus HCV QS-RGQ (Qiagen, Hilden Germany). Only high-quality serum samples, meeting our inclusion criteria, were considered for further analysis, specifically 22 out of the 37 patients, with six cases providing both pre- and post-treatment samples, resulting in a total of 28 patients (Table 1). The collected serum samples were stored frozen at the National Liver Institute Sustainable Sciences Institute Collaborative Research Centre (NLISSICRC).

Inclusion criteria for this study were as follows: availability of sufficient high-quality serum for viral titer assessment, documented relapse after primary treatment confirmed by qRT-PCR, and viral loads exceeding 1 × 10^5^ International Units (IU) as amplification of samples with lower viral loads could produce duplicate sequences, introducing bias in the detection of viral population mutations rates. Biobanking and viral load assessment was carried out at the NLISSICRC. Patients adhered to a daily regimen of medications specified by the National Committee for viral hepatitis control (NCCVH), which included Sofosbuvir (SOF) (400 mg), Ombitasvir (OMB) (25 mg), and Paritaprevir (150 mg). Ribavirin (RBV) dosage was adjusted according to the patient’s weight and cirrhosis status. The cohort consisted of 12 males and 10 females, aged 23 to 67 years (Table 1). Samples were sent frozen to the Vall d’Hebron Institut of Research (VHIR) at the Vall d’Hebron Barcelona Hospital Campus for NGS analysis.

HCV-RNA was manually extracted from 140 µL of serum using a QIAmp Viral RNA Mini Kit (Qiagen, Hilden, Germany). All samples were subjected to high-resolution HCV subtyping based on next-generation sequencing (NGS) of the NS5B region [17]. This high-throughput methodology allows accurate classification of subtypes and enables the design of subtype-specific primers (Table 3), minimizing bias when amplifying the regions of interest targeted by DAAs. Therefore, we amplified the three DAA-targeted genomic regions of HCV (NS3, NS5A, and NS5B) [22,29] using a two-step RT-PCR-Nested amplification protocol [22,30]. The nucleotide and amino acid positions were referenced using the full-length genome sequence H77 (accession number AF009606) [30]. The amplified products were deep sequenced using the Illumina MiSeq platform (Illumina, San Diego, CA, USA). An in-house R script was used to analyze the raw data and obtain a report of all substitutions present at the three targeted regions [18,22,29]. Quality filters for NGS reads were as follows: First, paired-end reads were overlapped with Flash, setting a minimum overlap of 20pb and a maximum of 10% differences, obtaining reads fully covering the amplicon. Next, reads with over 5% bp with a Phred score below Q30 were filtered out ensuring high quality in the reads and full coverage of the amplicon [3,18,29].

## 5. Conclusions

This study contributes novel insights by including a substantial number of samples from rarer HCV subtypes G4m and G4o. Our findings suggest that HCV subtypes may influence the pattern of RASs in the NS5A region following DAA-treatment failure. Deep sequencing techniques are crucial for accurate subtype classification and RASs identification, aiding in the design of effective rescue treatments.

## Figures and Tables

**Table 1:** Sample clinical description and cases pre/post-treatment identification. Abbreviations: DCV, Daclatasvir; SOF, Sofosbuvir; VEL, Velpatasvir; VOX, Voxilaprevir; RBV, Ribavirin; PAR, Paritaprevir; OMB, Ombitasvir.

Samples	Case	Age	Gender	Subtype	Viral Load (IU)	First Treatment	Treatment Period	Second Treatment. Final Decision
R001 Pre & R002 Post	1	54	Female	4a	5.80 × 10^5^ 3.51 × 10^6^	SOF/DCV	12 weeks	SOF/VEL/VOX
R007 Pre & R039 Post	3	23	Male	4a	5.49 × 10^6^ 1.95 × 10^6^	SOF/DCV	12 weeks	SOF/VEL/VOX
R010 Pre & R009 Post	4	60	Male	4a/4o	2.23 × 10^7^ 9.17 × 10^6^	SOF/DCV	12 weeks	SOF/VEL/VOX
R015 Pre & R016 Post	5	65	Male	4o	7.31 × 10^5^ 7.01 × 10^5^	SOF/DCV	12 weeks	SOF/VEL/VOX
R036 Pre & R035 Post	6	46	Female	4m	5.82 × 10^6^ 1.16 × 10^6^	SOF/DCV	12 weeks	SOF/VEL/VOX
R037		64	Male	4a	3.70 × 10^5^	SOF/DCV	12 weeks	SOF/VEL/VOX
R038		53	Male	4a	2.00 × 10^6^	SOF/DCV	12 weeks	SOF/VEL/VOX
R043		49	Male	4m	2.88 × 10^5^	SOF/DCV	12 weeks	SOF/VEL/VOX
R033		51	Female	4a	1.74 × 10^6^	SOF/DCV	12 weeks	SOF/VEL/VOX
R034		62	Male	4o	1.12 × 10^6^	SOF/DCV	12 weeks	SOF/VEL/VOX
R027		47	Female	4a	6.32 × 10^6^	SOF/DCV	12 weeks	SOF/VEL/VOX
R025		56	Male	4m	1.44 × 10^7^	SOF/DCV	12 weeks	SOF/VEL/VOX
R008		58	Female	4a	9.46 × 10^5^	SOF/DCV	12 weeks	SOF/VEL/VOX
R019		60	Male	4o	5.76 × 10^5^	SOF/DCV	12 weeks	SOF/VEL/VOX
R022		49	Female	4o	4.66 × 10^6^	SOF/DCV	12 weeks	SOF/VEL/VOX
R029		49	Female	1g	4.39 × 10^6^	SOF/DCV/RBV	12 weeks	SOF/VEL/VOX
R031		53	Male	4o	4.24 × 10^6^	SOF/DCV/RBV	12 weeks	SOF/VEL/VOX
R006 Pre & R005 Post	2	60	Female	4a	1.89 × 10^6^ 1.76 × 10^5^	SOF/PAR/OMB/RBV	24 weeks	SOF/VEL/VOX
R013		42	Female	4a	2.91 × 10^6^	SOF/PAR/OMB/RBV	24 weeks	SOF/VEL/VOX
R021		67	Male	4o	2.86 × 10^6^	SOF/PAR/OMB/RBV	24 weeks	SOF/VEL/VOX
R026		60	Male	4a	1.26 × 10^6^	SOF/PAR/OMB/RBV	24 weeks	SOF/VEL/VOX
R024		45	Female	4a	6.57 × 10^6^	SOF/PAR/OMB/RBV	24 weeks	SOF/VEL/VOX

**Table 2:** Number of post-treatment patients showing prevalent mutations in the cohort. (**a**) refers to substitutions with frequencies higher or equal to 50% found in six or more patients, whilst (**b**) refers to substitutions observed in positions that have been previously associated with resistance (RAS), in any number of patients in the cohort.

**(a)**	**HCV Region/Position**	**Amino Acid Substitution**	**No. of Patients (%)**	**Amino Acid Position**
Substitutions with frequencies >= to 50% in six or more patients	NS3	K92R	7	92
	NS5A	L28M	8	28
	NS5A	L30S	7	30
	NS5A	K44R	7	44
	NS5B1	N180K	7	180
	NS5B1	K184E	12	184
	NS5B1	A207S	11	207
	NS5B1	Q209K	11	209
	NS5B1	A254T	9	254
	NS5B2	D272E	6	272
**(b)**	**HCV Region**	**Amino Acid Substitution**	**Num. Patients**	**RAS Position**
Substitutions with frequencies >= to 50% at RAS positions	NS3	T54S	1	54
	NS3	T122S	2	122
	NS3	V170I	3	170
	NS5A	L28M	8	28
	NS5A	L28T	1	28
	NS5A	L30S	7	30
	NS5A	L30R	1	30
	NS5A	E62D	1	62
	NS5A	E62N	1	62
	NS5A	E62Q	1	62

**Table 3:** Specific RT-PCR-Nested primers for DAA-targeted regions.

	Genotype	Region	Oligo	5′ Position	Sequence
RT-PCR	G4a	NS3	Fw	3391	CAGAAACATCMAAGGGGTGGAGACT
			Rv	4004	CTGRGGCACYGCDGGGGGTGT
		NS5A	Fw	6230	GATCAATGAAGATTGYTCCACYCCAT
			Rv	6879	GTGATGGGTCTGTCARCATGGA
		NS5B	Fw	7952	CCACATCARCTCCGTGTGG
			Rv	8650	GGGGGAGCCGAGTAYCTCGT
	G4m/G4o	NS3	Fw	3412	TGGAGRCTYCTYGCYCCYAT
			Rv	4064	ACYTTRGTGCTCTTGCCGCT
		NS5A	Fw	6217	AGACGYCTYCACMAGTGGATYAAYGA
			Rv	6989	CABGTGGCYTTCARDGATGGRGC
		NS5B	Fw	7952	CCACATCARCTCCGTGTGG
			Rv	8631	TCATRGCCTCCGTGAAGGC
	G1g	NS3	Fw	3354	CGCAGGGGYAGGGAAGT
			Rv	4008	AGGTCTGRGGYACGGCTG
		NS5A	Fw	6213	AGGCGACTYCACACRTGGAT
			Rv	6879	GTGATGGRTCTGTGAGCATRGACG
		NS5B	Fw	7952	CCACATCAACTCCGTGTGG
			Rv	8638	GGGRGCGGAGTACCTGGT
Nested	G4a	NS3	Fw	3490	GGGACACCAATGARAATTGTGGT
			Rv	3982	GARTTGTCAGTGAACACTGGTGATC
		NS5A	Fw	6299	CGTGCTGAGTGACTTCAAGACGTGGCT
			Rv	6735	GGTGTAGYCTGAYGCCGTCYA
		NS5B1	Fw	7952	CCACATCARCTCCGTGTGG
			Rv	8409	CCCACRTAGAGTCTYTCTGTGAG
		NS5B2	Fw	8254	CNTAYGAYACCMGNTGYTTTGACTC
			Rv	8641	GARTAYCTGGTCATAGCNTCCGTGAA
	G4m/G4o	NS3	Fw	3481	AGCCTYACYGGCARRGAYACCAATG
			Rv	3983	TTGTCRGTRAAVACYGGRGACCTCAT
		NS5A	Fw	6288	TGGGTYTGCACYGTHYTRAGTGACT
			Rv	6811	CCVACYACRAAMGWGTTGAGGCC
		NS5B1	Fw	7952	CCACATCARCTCCGTGTGG
			Rv	8409	TGCTGTTRTACATSGGRCCRCC
		NS5B2	Fw	8254	CNTAYGAYACCMGNTGYTTTGACTC
			Rv	8641	GARTAYCTGGTCATAGCNTCCGTGAA
	G1g	NS3	Fw	3486	GGCCGVGAYAAMAACMMGGTGG
			Rv	3988	GGGGTRGAATTGTCMGTGWAHACDGG
		NS5A	Fw	6282	TGGGACTGGAHTGCAYGGT
			Rv	6729	GGCGYACCCCRTCYASCT
		NS5B1	Fw	7952	CCACATCAACTCCGTGTGG
			Rv	8389	CCGACGTACARTCTCTCRGTGAG
		NS5B2	Fw	8254	CNTAYGAYACCMGNTGYTTTGACTC
			Rv	8641	GARTAYCTGGTCATAGCNTCCGTGAA

## Data Availability

The genomic nucleotide sequences included in this study have been deposited into GenBank Sequence Read Archive database with the project number “PRJNA1036116”.

## Supplementary Materials

The following supporting information can be downloaded at: https://www.mdpi.com/article/10.3390/ijms262110649/s1.

## Abbreviations

VHIR	Vall d’Hebron Institut de Recerca
DAAs	Direct-Acting Antivirals
HCV	Hepatitis C Virus
RAS	Resistance-Associated Substitutions
SVR	Sustained Virologic Response
DCV	Daclatasvir
EBR	Elbasvir
LDV	Ledipasvir
OMB	Ombitasvir
PTV/r	Paritaprevir/Ritonavir
RBV	Ribavirin
SOF	Sofosbuvir
