# Supplementary material for: Deep Sequencing Analysis of Hepatitis C Virus Subtypes and Resistance-Associated Substitutions in Genotype 4 Patients Resistant to Direct-Acting Antiviral (DAA) Treatment in Egypt
Source: Int J Mol Sci. 2025 Oct 31;26(21):10649. doi: 10.3390/ijms262110649 (PMC12609098; doi:10.3390/ijms262110649)
Supplement: Supplementary file 1 [file ijms-26-10649-s001.zip › ijms-3920054-supplementary.pdf]

Supplementary Table S1. Number of reads obtained by each genotype HCV region (NS3, NS5A and two amplicons of NS5B 1 and 2. Subtype classification and the nucleotide position of each amplified region. Var refers to amino acid substitutions, with the reads supporting each substitution and PCT is the percentage of the mutation referred to the total number of reads obtained.

G4a

Sample ID	Total Reads	Region	Ref. genotype position	Var	Reads	Pct
R001	38126	NS3	G4a.3490.3982	K92R	38126	100
	93569	NS5A	G4a.6299.6735	**L28M**	**93569**	**100**
				**L30S**	**93569**	**100**
				K44R	93569	100
				E62N	92860	99,2
				P97A	971	1
				D126E	93569	100
				Q135A	93569	100
	99369	NS5B1	G4a.7952.8409	N131D	99354	100
				A146V	99354	100
				K184E	99354	100
				A207S	99354	100
				Q209K	99354	100
				T235V	99354	100
				V252A	99354	100
	61163	NS5B2	G4a.8254.8641	A235V	61163	100
				V252A	60953	99,7
				T254A	61163	100
				E258*	1212	2
				D272E	61163	100
				R304K	61163	100
				D310G	1357	2,2
				N333K	61163	100
				R337G	61163	100
R002	41833	NS3	G4a.3490.3982	V71I	555	1,3
				A95T	6306	15,1
				A101S	634	1,5
	66598	NS5A	G4a.6299.6735	**L28M**	**66385**	**99,7**
				K44R	66598	100
				V52I	3539	5,3
				T56K	3752	5,6
				E62N	62049	93,2
				E62D	3752	5,6
				D126E	66385	99,7
				Q135A	66385	99,7

	20903	NS5B1	G4a.7952.8409	N131D	20903	100
				P135S	20903	100
				A146V	20903	100
				K184E	20903	100
				A207S	20903	100
				Q209K	20903	100
				T235V	20903	100
	67321	NS5B2	G4a.8254.8641	A235V	67321	100
				V252A	7869	11,7
				T254A	67321	100
				N268S	1888	2,8
				D272E	66803	99,2
				Y296C	835	1,2
				R304K	66747	99,1
				N333K	67321	100
				R337G	67321	100
R005	27780	NS3	G4a.3481.3983	V33M	27780	100
				I48V	27780	100
				T54S	27780	100
				K92R	27780	100
				T95A	27780	100
				A101S	27780	100
				A129P	27780	100
				V170I	27780	100
	110529	NS5A	G4a.6299.6735	**L30S**	**5109**	**4,6**
				L37F	110189	99,7
				K44R	110529	100
				T56K	110529	100
				E62D	110529	100
				K68R	110529	100
				T75I	110529	100
				**Y93H**	**110529**	**100**
				V99A	3141	2,8
				F108V	110529	100
				F127S	1433	1,3
				K139R	1179	1,1
	106149	NS5B1	G4a.7952.8409	P135S	106149	100
				A146V	106149	100
				K184E	106149	100
				A207S	106149	100
				Q209K	106149	100
				T235V	106149	100
				V252A	106149	100
				A254T	106149	100
	71898	NS5B2	G4a.8254.8641	I233T	1198	1,7
				A235V	71632	99,6

				V252A	71898	100
				K270R	71655	99,7
				G275E	4924	6,8
				S288N	11270	15,7
				T294A	734	1
				L336P	805	1,1
R006	72888	NS3	G4a.3490.3982	K92R	72888	100
				A101S	72631	99,6
				I153L	72888	100
				V170I	72888	100
	106816	NS5A	G4a.6299.6736	L37F	101810	95,3
				K44R	106816	100
				T56K	106816	100
				E62D	106816	100
				I67V	1137	1,1
				T75I	106816	100
				I138V	1127	1,1
	41460	NS5B1	G4a.7952.8410	P135S	41460	100
				A146V	41460	100
				K184E	41460	100
				A207S	41460	100
				Q209K	41460	100
				T235V	41460	100
				V252A	41460	100
				A254T	41460	100
	97789	NS5B2	G4a.8254.8642	D232N	1351	1,4
				A235V	97789	100
				K251R	18417	18,8
				V252A	97789	100
				N333S	6337	6,5
R007	56055	NS3	G4a.3490.3982	K92R	56055	100
	81132	NS5A	G4a.6299.6736	**L28M**	**81132**	**100**
				**L30S**	**81132**	**100**
				Y43F	81132	100
				K44R	81132	100
				I67V	81132	100
				F108T	81132	100
	63304	NS5B1	G4a.7952.8410	I179V	63304	100
				N180K	63304	100
				K184E	63304	100
				T206N	63304	100
				A207S	63304	100
				Q209K	63304	100
				T213V	63304	100
				T235A	63304	100
				E237A	63304	100

				E248D	63304	100
				A254T	63304	100
	111658	NS5B2	G4a.8254.8642	K231R	1844	1,7
				E237A	111658	100
				E248D	111658	100
				E258G	2031	1,8
				D272E	111253	99,6
				I303T	111658	100
R008	26406	NS3	G4a.3490.3982	T46S	26406	100
				V48I	26406	100
				V71I	26406	100
				K92R	26406	100
				A101S	26406	100
				V170I	26406	100
	66847	NS5A	G4a.6299.6736	**L28M**	**66847**	**100**
				**L30S**	**66847**	**100**
				I34V	66364	99,3
				D50E	66847	100
				V52I	66847	100
				T56V	66847	100
				I67V	66847	100
				V130I	66847	100
	63540	NS5B1	G4a.7952.8410	R173K	63540	100
				I179V	63540	100
				N180K	63540	100
				K184E	63540	100
				F203Y	63540	100
				A207S	63540	100
				Q209K	63540	100
				T235V	63540	100
				A254T	63540	100
	136307	NS5B2	G4a.8254.8642	A235V	135889	99,7
				K251R	1592	1,2
				E258G	3615	2,7
				Y285F	135694	99,6
				R304K	135842	99,7
R010	32731	NS3	G4a.3490.3982	P89S	7273	22,2
				K92R	32731	100
				A151V	32731	100
	82170	NS5A	G4a.6299.6736	R41K	14190	17,3
				K44R	82170	100
				I67V	82170	100
				A103T	19967	24,3
				V130I	14352	17,5
	10901	NS5B1	G4a.7952.8410	E124K	138283	100
				P156A	138283	100

				H176Y	138283	100
				I179V	138283	100
				N180R	136585	98,8
				N180K	1698	1,2
				K184E	138283	100
				A207S	138283	100
				Q209K	138283	100
				T235A	138283	100
				K251R	1580	1,1
				V252A	77968	56,4
	134229	NS5B2	G4a.8254.8642	K251R	1549	1,2
				V252A	112650	83,9
				T254A	131196	97,7
				D272E	134229	100
				I303V	122205	91
				V322I	133628	99,6
R013	55413	NS3	G4a.3490.3982	T122A	7599	13,7
				K92R	55413	100
				A151V	55413	100
				V170I	55413	100
	75910	NS5A	G4a.6299.6736	**L28M**	**75910**	**100**
				**L30S**	**75910**	**100**
				K44R	75910	100
				T56V	75910	100
				E62Q	75910	100
				V130I	75910	100
	61805	NS5B1	G4a.7952.8410	P135S	61805	100
				N180K	61805	100
				K184E	61805	100
				A207S	61805	100
				Q209K	61805	100
				T235V	61805	100
				A254T	61805	100
	117250	NS5B2	G4a.8254.8642	A235V	117250	100
				D272E	116839	99,6
				R304K	117250	100
				A338V	62712	53,5
R024	23122	NS3	G4a.3481.3983	I48V	23122	100
				S61G	23122	100
				T63S	23122	100
				N72C	23122	100
				T95A	23122	100
				Y105F	23122	100
				V107I	23122	100
				T122S	23122	100
				A129P	23122	100

				M147L	23122	100
				A151V	23122	100
				L153I	23122	100
	73423	NS5A	G4a.6299.6736	**L28M**	**64037**	**87,2**
				**L30S**	**64037**	**87,2**
				I34V	20650	28,1
				K44R	9386	12,8
				V52I	64037	87,2
				S71T	64037	87,2
				I101V	4233	5,8
				N105D	63867	87
				F108S	4429	6
				D126E	63993	87,2
				V130I	9430	12,8
	26847	NS5B1	G4a.7952.8410	N131I	26847	100
				N180K	26847	100
				K184E	26847	100
				A207S	26847	100
				Q209K	26847	100
				T213V	26847	100
				A254T	26847	100
	101620	NS5B2	G4a.8254.8642	A235T	101143	99,5
				K270R	1538	1,5
				D272E	101260	99,6
				Y276N	101620	100
				R309K	101310	99,7
				N333A	101620	100
R026	24966	NS3	G4a.3490.3982	A87T	372	1,5
				A95T	24641	98,7
				L175P	403	1,6
	89806	NS5A	G4a.6299.6735	**L28M**	**42307**	**47,1**
				**L28T**	**47041**	**52,4**
				T56I	10134	11,3
				R73K	12455	13,9
				T83M	89806	100
				F127S	1226	1,4
				V130I	89806	100
	22216	NS5B1	G4a.7952.8409	K184E	22216	100
				A207S	22216	100
				Q209K	22216	100
				T235A	22216	100
				E237A	22216	100
				A254T	22216	100
	43825	NS5B2	G4a.8254.8641	A235V	590	1,3
				E237A	43235	98,7
				T300S	43235	98,7

				I303V	729	1,7
				R309K	590	1,3
				D327G	3073	7
				R337Q	43084	98,3
R027	33896	NS3	G4a.3490.3982	K92R	33757	99,6
				A150V	33757	99,6
	73958	NS5A	G4a.6299.6735	**L28M**	**73958**	**100**
				**L30S**	**73958**	**100**
				T56R	73958	100
				V130I	73958	100
	27793	NS5B1	G4a.7952.8409	P135S	27793	100
				N180K	27793	100
				K184E	27793	100
				A207S	27793	100
				Q209K	27793	100
				T235V	27793	100
				A254T	27793	100
	244682	NS5B2	G4a.8254.8641	A235V	243778	99,6
				**E237G**	**2491**	**1**
				C242R	3724	1,5
				K251R	2926	1,2
				I303T	2950	1,2
				R309K	244682	100
R033	35956	NS3	G4a.3490.3982	T122S	35956	100
				S133A	35771	99,5
	55700	NS5A	G4a.6299.6735	**L28M**	**55700**	**100**
				**L30S**	**55700**	**100**
				K44R	55700	100
				R123K	55700	100
				V124L	55700	100
				V130I	55489	99,6
	41979	NS5B1	G4a.7952.8409	A140V	433	1
				V161A	495	1,2
				V178I	41979	100
				N180S	41979	100
				Q181L	1482	3,5
				L182Q	1482	3,5
				K184E	41979	100
				V186M	1142	2,7
				S196A	450	1,1
				A207S	41979	100
				Q209K	41979	100
				K251R	581	1,4
				V252A	41979	100
				A254T	41979	100
	224659	NS5B2	G4a.8254.8641	A235T	224659	100

				V252A	224659	100
				H267Y	224659	100
				N333S	223852	99,6
R037	6783	NS3	G4a.3481.3983	L44M	6783	100
				I48V	6783	100
				T54S	6783	100
				V114I	6783	100
				A129P	6783	100
				L153I	6783	100
	70769	NS5A	G4a.6299.6735	L30R	70769	100
				I34V	70769	100
				D126E	70769	100
	123383	NS5B1	G4a.7952.8389	E124G	1981	1,6
				D125N	3624	2,9
				N130S	123383	100
				N131D	2815	2,3
				P135S	123383	100
				N142H	1639	1,3
				Y162F	123383	100
				N180K	123383	100
				K184E	123383	100
				F203Y	123383	100
				S207A	123383	100
				K212R	123383	100
				T213N	123383	100
				A249V	2978	2,4
	135015	NS5B2	G4a.8254.8641	V252A	4904	3,6
				K270R	3860	2,9
				F289L	4544	3,4
				I303T	135015	100
R038	30214	NS3	G4a.3481.3983	I48V	30214	100
				K92R	30214	100
				T95A	30214	100
				A129P	30214	100
				L153I	30214	100
	54022	NS5A	G4a.6299.6735	**L28M**	**54022**	**100**
				**L30S**	**54022**	**100**
				Y43F	54022	100
				K44R	54022	100
				I67V	54022	100
				F108T	54022	100
	24415	NS5B1	G4a.7952.8409	I179V	24415	100
				N180K	24415	100
				K184E	24415	100
				T206N	24415	100
				A207S	24415	100

				Q209K	24415	100
				T213V	24415	100
				T235A	24415	100
				E237A	24415	100
				E248D	24415	100
				A254T	24415	100
	212866	NS5B2	G4a.8254.8641	E237A	212866	100
				E248D	212081	99,6
				K251R	4318	2
				D272E	212866	100
				Y285H	2161	1
				I303T	212866	100
R039	38411	NS3	G4a.3481.3983	K92R	38411	100
				A95T	474	1,2
	76980	NS5A	G4a.6299.6735	**L28M**	**76980**	**100**
				**L30S**	**76980**	**100**
				Y43F	76980	100
				K44R	76980	100
				I67V	76980	100
				F108T	76980	100
				V130I	881	1,1
	64742	NS5B1	G4a.7952.8409	E124G	2036	3,1
				I134M	1768	2,7
				I179V	64742	100
				N180K	64742	100
				K184E	64742	100
				L204P	852	1,3
				T206N	63836	98,6
				T206S	906	1,4
				A207S	64742	100
				Q209K	64742	100
				T213V	64742	100
				T235A	64524	99,7
				E237A	64742	100
				E248D	64742	100
				A254T	64742	100
	217018	NS5B2	G4a.8254.8641	E237A	217018	100
				E248D	217018	100
				D272E	217018	100
				I303T	217018	100
				T312A	6021	2,8

G4o/l/m

Sample ID	Total Reads	Region	Ref. genotype position	Var	Reads	Pct
R009	10597	NS3	G4o.3481.3983	T95A	10597	100
				T98A	10597	100
	58445	NS5A	G4o.6288.6811	**T30S**	**58445**	**100**
				D62E	58445	100
				L64A	58047	99,3
				V67I	58445	100
				K107E	58445	100
	102033	NS5B1	G4o.7952.8409	A130T	164985	100
				V131T	164985	100
				T184G	164985	100
				A207T	1696	1
				S210A	2425	1,5
				K211R	1784	1,1
				N244D	162962	98,8
	82443	NS5B2	G4o.8254.8641	N244D	117914	100
R015	46518	NS3	G4o.3481.3983	M52L	46518	100
				P67S	46518	100
				T92A	46518	100
				L127I	46518	100
				R130K	46518	100
				S133G	46518	100
				T134L	46518	100
	63456	NS5A	G4o.6288.6811	K20R	63456	100
				**T30S**	**63456**	**100**
				R41K	63146	99,5
				F43Y	63456	100
				T56I	63456	100
				D62N	63456	100
				L64A	63456	100
				K68R	63456	100
				I74L	63456	100
				G98S	63456	100
				V99I	63456	100
				N105S	63456	100
				M133V	63456	100
				S176T	63456	100
	53549	NS5B1	G4o.7952.8409	E124G	600	1,1
				A130T	53549	100
				V144A	1063	2
				T184R	53549	100
				F193S	1587	3
				E202S	53549	100

				L205S	3550	6,6
				A206N	53549	100
				W208*	2187	4,1
				R209Q	53549	100
				V213N	3484	6,5
				V213T	50065	93,5
				N244D	53549	100
	150158	NS5B2	G4o.8254.8641	C242R	2371	1,6
				N244D	150158	100
				Q276V	149521	99,6
				T329V	149683	99,7
				E331G	1630	1,1
R016	56051	NS3	G4o.3481.3983	M52L	56051	100
				P67S	56051	100
				T92A	56051	100
				L127I	56051	100
				R130K	56051	100
				S133G	56051	100
				T134L	56051	100
	54270	NS5A	G4o.6288.6811	K20R	54270	100
				**T30S**	**54270**	**100**
				R41K	53786	99,1
				F43Y	54270	100
				T56I	54270	100
				D62N	54270	100
				L64A	54270	100
				K68R	54270	100
				I74L	54270	100
				G98S	54270	100
				V99I	54270	100
				N105S	54270	100
				M133V	54270	100
				S176T	54270	100
	39385	NS5B1	G4o.7952.8409	E124G	724	1,8
				A130T	39385	100
				K151R	1653	4,2
				T184R	39385	100
				E202S	39385	100
				L204F	493	1,3
				A206N	38721	98,3
				A206D	664	1,7
				R209Q	39385	100
				V213T	39385	100
				V239I	1090	2,8
				N244D	39385	100
	98684	NS5B2	G4o.8254.8641	N244D	98684	100

				M266I	1632	1,7
				Y267H	2352	2,4
				Q276V	98684	100
				K298R	1302	1,3
				T329V	98124	99,4
R019	74475	NS3	G4o.3481.3983	M74L	74475	100
				T95A	74475	100
				T98S	74475	100
	65662	NS5A	G4o.6288.6811	**T30S**	**65662**	**100**
				D62N	65662	100
				L64A	65662	100
				V67I	65662	100
				V99I	65662	100
				E126D	65662	100
				M133V	65662	100
				S176T	65662	100
	56379	NS5B1	G4o.7952.8409	E124K	55607	98,6
				A140T	1422	2,5
				N142T	56379	100
				K151R	56379	100
				K173R	56379	100
				A178P	56379	100
				T184R	56379	100
				K211R	984	1,7
				V213E	56379	100
				N244D	56379	100
	68915	NS5B2	G4o.8254.8641	G234A	1534	2,2
				N244D	67381	97,8
				F285Y	909	1,3
				S288G	1027	1,5
				K309R	944	1,4
				A333I	66220	96,1
				Q334R	66220	96,1
				R337Q	66809	96,9
R021	54637	NS3	G4o.3481.3983	T92S	54637	100
				T95A	54637	100
				I153V	54637	100
	48237	NS5A	G4o.6288.6811	**T30S**	**48237**	**100**
				**M31V**	**3808**	**7,9**
				R41K	47975	99,5
				D62N	48237	100
				L64A	48237	100
				V67I	48237	100
				I101V	48237	100
				S174C	45237	93,8
	53870	NS5B1	G4o.7952.8409	E124K	40379	75

				E128G	13014	24,2
				A130T	53870	100
				N142T	40722	75,6
				T184G	53870	100
				A189T	53870	100
				A206T	40482	75,1
				G234A	53870	100
	113158	NS5B2	G4o.8254.8641	G234A	113158	100
				K309R	67222	59,4
				V315A	2901	2,6
				R337Q	113158	100
R022	23751	NS3	G4o.3481.3983	P89S	23751	100
				T92A	23751	100
				T95A	23751	100
	38010	NS5A	G4o.6288.6811	R41K	618	1,6
				T56I	38010	100
				D62N	4817	12,7
				D62T	33193	87,3
				L64A	38010	100
				V67I	37668	99,1
				**Y93H**	**37166**	**97,8**
	52172	NS5B1	G4o.7952.8409	E124G	566	1,1
				A130T	51969	99,6
				V131T	51876	99,4
				N244D	52172	100
				V252A	943	1,8
	129700	NS5B2	G4o.8254.8641	N244D	129700	100
				V252A	2576	2
				F285Y	129700	100
R025	58433	NS3	G4m.3481.3983	V71I	58433	100
				I107V	58433	100
				I114V	58433	100
	37135	NS5A	G4m.6288.6811	F37L	37135	100
				K44R	37135	100
				M72I	37135	100
				Q85H	37135	100
				A114S	37135	100
				D171E	37135	100
	55250	NS5B1	G4m.7952.8409	E124A	54925	99,4
				G128E	55250	100
				I131N	55250	100
				E206D	55250	100
				V235I	55250	100
				V235I	115096	99,7
	115444	NS5B2	G4m.8254.8641	R270K	114926	99,6
				Y276V	115444	100

				Y296H	1175	1
				R304K	115444	100
R031	51726	NS3	G4o.3481.3983		no subs.	
	67191	NS5A	G4o.6288.6811	H54Y	67191	100
				T56I	67191	100
				D62N	67191	100
				L64A	67191	100
				V67I	67191	100
				**Y93N**	**67191**	**100**
				V156I	18915	28,2
	49172	NS5B1	G4o.7952.8409	E124G	680	1,4
				A130T	49172	100
				V131T	49172	100
				S146A	49172	100
				T184G	49172	100
				E202D	49172	100
				N244D	49172	100
				V252A	49172	100
	172450	NS5B2	G4o.8254.8641	N244D	169253	98,1
				V252A	172450	100
				Q276M	172450	100
				A324T	2852	1,7
R034	25424	NS3	G4o.3481.3983	I48V	25424	100
				T63S	25424	100
				C65S	25424	100
				N72C	25424	100
				T92K	25424	100
				T95A	25424	100
				I114V	25424	100
				T122S	25424	100
				Q147L	25424	100
	53385	NS5A	G4o.6288.6811	L37V	17954	33,6
				R41K	53385	100
				K44R	3670	6,9
				V52I	2518	4,7
				T56K	7407	13,9
				D62N	45978	86,1
				L64A	53385	100
				V67I	53385	100
				**Y93C**	**16050**	**30,1**
				**Y93H**	**30215**	**56,6**
				**Y93S**	**4720**	**8,8**
				K164Q	53385	100
				L168M	53385	100
	37573	NS5B1	G4o.7952.8409	E124G	600	1,6
				A130G	37573	100

				L159F	762	2,0
				C170R	428	1,1
				K181N	444	1,2
				T184R	37573	100
				N244D	37573	100
				I253T	758	2,0
	232342	NS5B2	G4o.8254.8641	E238A	9248	4,0
				N244D	229368	98,7
				N244G	2974	1,3
				A324T	2610	1,1
				D332G	3652	1,6
R035	25693	NS3	G4m.3481.3983	I64L	25693	100
	81360	NS5A	G4m.6288.6811	**M31V**	**81360**	**100**
				F37L	81360	100
				K56Q	81360	100
				G65D	944	1,2
				Q85H	81360	100
				V99M	81360	100
				I101V	81360	100
				F108S	81360	100
				A114S	81360	100
				T137A	81360	100
				S146A	81360	100
				V156I	81360	100
				T176M	81360	100
	42281	NS5B1	G4m.7952.8409	E124G	872	2,1
				G128E	42281	100
				I131T	42281	100
				M139I	1908	4,5
				S146P	42281	100
				P156S	42281	100
				A207T	10868	25,7
				F217S	2671	6,3
				A255S	42281	100
	185957	NS5B2	G4m.8254.8641	A255S	185364	99,7
				Y267H	5337	2,9
				R270K	185957	100
				F289L	185957	100
				T312M	2321	1,2
R036	3824	NS3	G4m.3481.3983	I64L	3824	100
	59964	NS5A	G4m.6288.6811	**M31V**	**59964**	**100**
				F37L	59553	99,3
				K56Q	59964	100
				Q85H	59964	100
				V99M	59964	100
				I101V	51972	86,7

				F108S	58968	98,3
				A114S	59964	100
				T137A	59964	100
				S146A	59964	100
				V156I	59964	100
				T176M	36542	60,9
	42207	NS5B1	G4m.7952.8409	G128E	42043	99,6
				N130S	600	1,4
				I131T	42207	100
				S146P	42207	100
				P156S	42207	100
				E206D	33167	78,6
				A255S	42207	100
	33069	NS5B2	G4m.8254.8641	A255S	32712	98,9
				R270K	32871	99,4
				F289L	33069	100
R043	4718	NS3	G4m.3481.3983	V48I	4718	100
				G61S	4718	100
				S65C	4718	100
				C72N	4718	100
				K92S	4718	100
				A95T	4718	100
				T98A	4718	100
				H110N	4718	100
				V114I	4718	100
				S122T	4718	100
				L147Q	4718	100
	83639	NS5A	G4m.6288.6811	F37L	83639	100
				K56R	83639	100
				S71T	83639	100
				**Y93N**	**83639**	**100**
				A114S	83639	100
				D171E	83639	100
				T176M	83639	100
	43088	NS5B1	G4m.7952.8409	E124G	622	1,4
				G128E	43088	100
				I131T	43088	100
				E206D	43088	100
				M215V	505	1,2
				V235I	43088	100
				I253M	646	1,5
				T254A	1299	3
	62156	NS5B2	G4m.8254.8641	V235I	61965	99,7
				Y240H	1059	1,7
				P247S	3358	5,4
				T249A	1364	2,2

				R270K	62156	100
				R278G	682	1,1
				Y296N	2692	4,3
				R304K	61965	99,7
				G327E	61965	99,7

G1g

Sample ID	Total Reads	Region	Ref. genotype position	Var	Reads	Pct
R029	19650	NS3	G1g.3486.3988	M36V	19650	100
				R67K	6270	31,9
				L110H	19650	100
				F169V	237	1,2
	122470	NS5A	G1g.6282.6729	L16M	122470	100
				R24S	97029	79,2
				A25S	25441	20,8
				**Q30R**	**97426**	**79,6**
				V52I	122470	100
				L74I	11635	9,5
				H85Y	95072	77,6
				T87S	122470	100
				T101A	122470	100
				R108C	104785	85,6
				V130I	1631	1,3
				A135T	102780	83,9
				C140R	3290	2,7
				Q143H	2716	2,2
				V144D	2716	2,2
	40365	NS5B1	G1g.7952.8389	L127P	427	1,1
				I179V	16792	100
				Q184L	16717	99,6
				E189A	16688	99,4
				L198A	16792	100
				T235M	16079	95,8
				T251V	16693	99,4
	185971	NS5B2	G1g.8254.8641	T235M	177959	95,7
				E236G	2155	1,2
				T251V	184266	99,1
				R270K	184488	99,2
				A301S	185971	100
				G307R	11874	6,4
				V327A	180352	97
				A333V	5438	2,9
				S335G	4166	2,2
